# A systematic review of the frequency and severity of manic symptoms reported in studies that compare phenomenology across children, adolescents and adults with bipolar disorders

**DOI:** 10.1186/s40345-017-0071-y

**Published:** 2017-02-03

**Authors:** Faye Ryles, Thomas D. Meyer, Jaime Adan-Manes, Iain MacMillan, Jan Scott

**Affiliations:** 1 Early Intervention in Psychiatry Hub, NTW NHS Trust, Newcastle upon Tyne, UK; 2 Department of Psychiatry and Behavioral Sciences, University of Texas, Houston, TX USA; 3 Department of Psychiatry, La Princesa Hospital, Madrid, Spain; 4 Academic Psychiatry, Wolfson Unit, Institute of Neuroscience, Campus for Ageing and Vitality, Newcastle University, Newcastle upon Tyne, UK

**Keywords:** Systematic review, Mania, Phenomenology, Children, Adolescents, Adults, Manic symptoms, Irritability, Activity, Cognition

## Abstract

**Background:**

In the last two decades, there has been a significant increase in the diagnosis of Bipolar Disorder (BD) in children. The notion of prepubertal onsets of BD is not without controversy, with researchers debating whether paediatric cases have a distinct symptom profile or follow a different illness trajectory from other forms of BD. The latter issue is difficult to address without long-term prospective follow-up studies. However, in the interim, it is useful to consider the phenomenology observed in groups of cases with different ages of onset and particularly to compare manic symptoms in children diagnosed with BD compared to cases presenting with BD in adolescence and adulthood. This review systematically explores the phenomenology of manic or hypomanic episodes in groups defined by age at onset of BD (children, adolescents and adults; or combined age groups e.g. children and adolescents versus adults).

**Methods:**

Literature reviews of PubMed and Scopus were conducted to identify publications which directly compared the frequency or severity of manic symptoms in individuals with BD presenting with a first episode of mania in childhood, adolescence or adulthood.

**Results:**

Of 304 studies identified, 55 texts warranted detailed review, but only nine studies met eligibility criteria for inclusion. Comparison of manic symptoms across age groups suggested that irritability is a key feature of BD with an onset in childhood, activity is the most prominent in adolescent-onset BD and pressure of speech is more characteristic of adult-onset BD. However, none of the eligible studies made a direct comparison of phenomenology in children versus adults. Assessment procedures varied in quality and undermined the reliability of cross-study comparisons. Other limitations were: the scarcity of comparative studies, the geographic bias (most studies originated in the USA), the failure to fully consider the impact of psychiatric comorbidities on recorded symptoms and methodological heterogeneity.

**Conclusions:**

Despite frequent discussion of similarities and differences in phenomenology of mania presenting in different age groups, systematic research is lacking and studies are still required to reliably establish whether the frequency and severity of manic symptoms varies. Such information has implications for clinical practice and the classification of mental disorders.

## Background

Bipolar Disorder (BD) is a severe mental disorder that involves changes in mood, cognition and behaviour. It can be divided into three broad subgroups: BD-I (characterized by episodes of mania and depression); BD-II (hypomania and depression) and a heterogeneous group that is sometimes referred to as ‘spectrum disorders’, which includes BD-NOS (Not Otherwise Specified), cyclothymia, and other less well-defined BD-like syndromes (Akiskal et al. 2000; American Psychiatric Association (APA) 2000, 2013). The worldwide prevalence of all manifestations of BD is about 4% (Angst 1988). The peak age of onset is 15–25 years, but the incidence remains quite high throughout early and mid-adult life (Merikangas et al. 2011). It is suggested that cases with adolescent or adult onset typically present with similar symptom profiles for each phase of the disorder e.g. manic, hypomanic, depressive and mixed episodes (where depressive and manic symptoms occur simultaneously), and that the frequency of different types of episodes are also comparable (e.g. depressive episodes are common; mixed states are relatively rare) (Angst 1988). There have been some variations reported in these characteristics by age of onset, but overall cases presenting in adolescence or adulthood are usually regarded as having ‘adult-pattern’ BD with distinct episodes (Carlson 2011; Merikangas et al. 2011; Douglas and Scott 2014).

In the last two decades, there has been a significant increase in the diagnosis of BD in childhood, the so-called paediatric or juvenile-onset form of BD (Moreno et al. 2007). The notion of prepubertal onsets of BD is not universally accepted, with researchers debating everything from whether the condition exists in this age group (or if it is a misdiagnosis of other childhood conditions such as Attention Deficit Hyperactivity Disorder (ADHD)) and, if it does exist, how common it is, etc. (Douglas and Scott 2014; James et al. 2014). Whilst researchers and clinicians do not deny that children diagnosed with paediatric BD have psychological problems that need care and treatment, there is no consensus on whether this childhood condition is the same disorder as ‘adult-pattern’ BD that typically presents from adolescence onwards (Carlson and Klein 2014; Wozniak et al. 2010; Serra et al. 2016). One issue that has fueled this debate is the lack of consensus on the core symptoms of hypomania or mania [which we will refer to as (hypo)mania] presenting in children. For example, several researchers suggest that the juvenile form of BD is more likely to present with irritability rather than elation in mania, that mixed states may be more common, and/or that there are differences in the frequency or severity of BD symptoms observed in prepubertal children compared to other age groups (Findling et al. 2001; Leibenluft et al. 2003; Geller et al. 2004; Youngstrom et al. 2008). This is an interesting and important idea but, many of the publications rely on reports of the frequency of specific (hypo)manic symptoms in samples comprised children only, rather than considering studies that directly compare the symptoms of (hypo)manic episodes across age groups. Furthermore, studies of phenomenology often use different approaches to measuring the symptoms. For example, some studies report the presence or absence of the specific symptoms listed in internationally agreed diagnostic criteria (such as the A and B criteria reported in the Diagnostic and Statistical Manual (DSM IV); APA, 2000). In contrast, other studies use symptom rating scales (such as the Young Mania Rating Scale; YMRS; Young et al. 1978), which assess the severity of any symptoms that are present (and report the mean severity score for each item on the rating scale). Lastly, some studies of children use information obtained from interviews with a parent (and/or a teacher), whilst studies of adolescents and adults usually primarily rely on information obtained from interviews with the index case (the person with BD) (Douglas and Scott 2014).

The primary purpose of this review is to explore systematically whether the clinical phenomenology of (hypo)mania differs across three age groups (children, adolescents and adults) or across younger versus older age groups (e.g. a combined group of children and adolescents compared to adults with BD). The specific research questions are:

1. Is there a difference in the most frequently reported symptoms of (hypo)mania in different age groups in comparative studies that use recognized diagnostic criteria, e.g. DSM (American Psychiatric Association 1980, 2000) or ICD (International Classification of Diseases; World Health Organization 1992), or that employ scales that measure the core symptoms of BD, e.g. Kiddie-Schedule for Affective Disorders and Schizophrenia (K-SADS; Endicott and Spitzer 1978)?
2. Is there a difference in which symptoms of (hypo)mania are rated as the most severe in different age groups in comparative studies that used established symptom-rating scales, e.g. the YMRS?

## Methods

To answer the key research questions, we identified publications that made a direct comparison of the symptoms of (hypo)mania in individuals with childhood, adolescent and/or adult-onset BD.

### Search strategy

A systematic search of two online databases (Scopus and PubMed) was undertaken to identify any potentially relevant peer-reviewed original articles, abstracts or conference proceedings. Citation lists of publications were also searched for additional publications. The time frame for the search was limited from January 1st 1980 until September 30th 2016. The start date was chosen because this was the first time the diagnosis of BD was included by the DSM classification system (DSM III; American Psychiatric Association 1980). The search used combinations of terms from three broad categories (see “Appendix” for details): group 1 used various terms for BD (e.g. manic depress*); group 2 included terms for age groups (e.g. juven*); and group 3 focused on terms used to describe manic or hypomanic symptoms (e.g. psychopathol*).

**Fig. 1:**
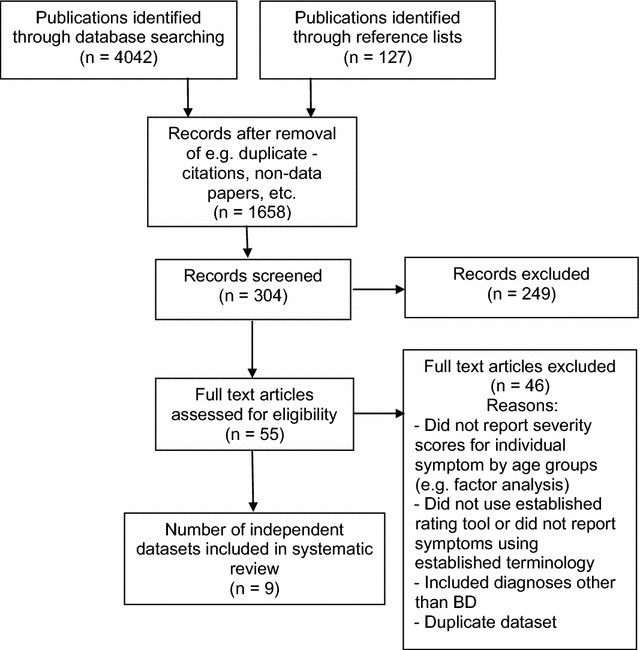
Study flow chart

The preliminary search was conducted by FR with consultations held with JS (e.g. if clarification was required regarding the eligibility of a study). The initial searches identified 1658 titles, of which 304 abstracts that were potentially relevant (see the flow chart provided in Fig. 1). Examination of abstracts identified that 55 full text publications warranted detailed examination.

### Eligibility criteria

The selected full text publications were assessed using the following eligibility criteria:

Inclusion criteria:

- Some or all study participants had a diagnosis of BD, and the data on BD cases were reported separately.
- The study reported a comparison of symptoms between at least two groups defined by age of onset and at least one of these groups comprised children, adolescents or adults only.
- The symptoms were reliably recorded using either recognized diagnostic criteria (assessed by clinical interview, case note review or a researcher using a diagnostic interview schedule) or an established symptom rating scale (e.g. K-SADS Mania Rating Scale (K-MRS); Kaufman et al. 1997).

Exclusion criteria:

- Studies where age at onset or age ranges included in any group were unclear.
- Studies that reported data for only one gender group (e.g. the sample was 100% male).
- Studies that did not report the raw data for the ratings of individual symptoms that were included in any group comparisons that were reported (e.g. some studies reported the items included in a factor analysis, but did not provide the mean scores for each item), or the information on symptom ratings could not be obtained from elsewhere (e.g. another publication from the same dataset or direct from the authors).
- Studies where symptoms were rated using idiosyncratic rating scales of unknown or uncertain reliability or validity, and/or the scales employed have not been used in any other studies of BD.
- Duplicate publications or additional publications from the same original dataset.
- Studies that were not written in English, French, Spanish or German.

### Data extraction and coding

The review was carried out following the Preferred Reporting Items for Systematic Reviews and Meta-Analyses (PRISMA) guidelines (Moher et al. 2009).

For studies meeting eligibility criteria, information was extracted on: number of participants, country and year of study, clinical setting, gender distribution, age groups examined and BD subtypes included (see Table 1). The quality of eligible studies was assessed using the Critical Appraisal Skills Programme Checklist for systematic reviews (CASP 2013), which considers a range of key criteria including population studied (sample size and representativeness), methodology and standard of reporting of statistical analysis.

**Table 1:** Sample characteristics for eligible publications (listed by year of publication)

Publication	Country	Sample size (*n*)	Gender (% males)^b^	Age groups (age range in years and number of participants per group)^a^			Setting	BD subtypes
				Child	Adolescent	Adult		
Ballenger et al. (1982)	USA	21	NK		<21 (*n* = 9)	>30 (*n* = 12)	Inpatient	BD-I (mania)
McElroy et al. (1997)	USA	128	43%		12–18 (*n* = 40)	19–45 (*n* = 88)	Inpatient	BD-I (mania)
Findling et al. (2001)	USA	90	71%	5–11 (*n* = 56)	12–17 (*n* = 34)		Outpatient	BD-I
Jerrell and Shugart (2004)	USA	267	52%	7–17 (*n* = 83)		18–59 (*n* = 184)	Inpatient and outpatient	BD-I
Lazaro et al. (2007)	Spain	43	40%	< 13 (*n* = 14)	≥13 (*n* = 29)		Outpatient	BD-I, BD-II and BD-NOS
Birmaher et al. (2009)^c^	USA	263	53%	4–11 (*n* = 173)	<12 (*n* = 90)		Inpatient and outpatient	BD-I, BD-II and BD-NOS
Song et al. (2010)	Korea	53	59%	NK (*n* = 16)	NK (*n* = 37)		Inpatient	BD-I, BD-II and BD-NOS
Chan et al. (2011)	UK	35	51%	7–12 (*n* = 9)	13–18 (*n* = 26)		Outpatient	BD-I, BD-II and BD-NOS
Safer et al. (2012)	USA	1106	NK	10–17 (*n* = 457)		18–65 (*n* = 649)	Inpatient and outpatient	BD-I (mania)

*NK* not known

^a^Age Groups: child refers to prepubertal children or those aged ≤12; adolescent refers to age ≥13 to 18 years, although one study extended the age range up to 21 years; –adult refers to individuals aged ≥18 years, although one study chose a minimum of age ≥30 years

^b^Percentages are reported to the nearest integer

^c^The study reported three age groups, but only two met eligibility criteria for inclusion in this review

Data from each eligible study were reviewed, and each publication was categorized by the age groups included. Three sub-sets were identified:

- studies that reported the proportion of the sample with one or more of the diagnostic symptoms of (hypo)mania;
- studies that reported the proportion of cases with symptoms assessed using a diagnostic interview schedule;
- studies that reported the mean scores for each symptom on a severity rating scale, or used another recognized approach to reporting the severity of symptoms, e.g. the percent of maximum possible item score (POMP).

### Data synthesis

FR identified the six most frequent (or for all the symptoms reported, if less than six were examined) or the six most severe symptoms reported in each age group included in each study. The symptom descriptions and rankings (as summarized in Tables 2 and 3) are described using the specific item descriptions provided in the original assessment scale and the frequency or severity data were as reported by the original researchers.

**Table 2:** Rank order of frequency of manic symptoms by age group (symptom frequencies are reported as  %)

Ballenger et al. (1982)		Findling et al. (2001)^a^		Jerrell and Shugart (2004)		Lazaro et al. (2007)^b^		Song et al. (2010)^c^		Chan et al. (2011)	
Adolescent	Adult	Child	Adolescent	Child/ Adolescent	Adult	Child	Adolescent	Child	Adolescent	Child	Adolescent
Grandiosity (78)	Pressured Speech (100)	Poor judgement (91)	Increased goal directed/ aggressive behaviour (88)	Irritable (83)	Irritable (74)	Irritability, aggression, anger outbursts (64)	Euphoria (35)	Irritability, aggressive behaviour (63)	Depression (49)	Elevated mood (89)	Elevated mood (73)
Decreased sleep (67)	Decreased sleep (100)	Racing thoughts (88)	Racing thoughts (88)	Easily distracted (49)	Racing thoughts (55)	Psychotic episode (7)	Psychotic episode (7)	Depression (19)	Irritability, aggressive behaviour (24)	Decreased concentration (44)	Decreased sleep (62)
Pressured Speech (67)	Hyperactivity (92)	Bizarre/ grandiose thought content (86)	Distractibility (88)	Euphoric (42)	Easily distracted (46)	Euphoria (0)	Irritability, aggression, anger outbursts (3)	Elated mood, euphoria (13)	Elated mood, euphoria (16)	Restlessness (33)	Decreased concentration (46)
Belligerence (67)	Euphoria (75)	More talkative (84)	Bizarre/ grandiose thought content (79)	Hardly slept, not tired (31)	Talked fast (45)			Mood swings, fluctuation (6)	Mood swings, fluctuation (11)	Decreased sleep (33)	Impulsivity (39)
Flight of ideas (44); Hyper-sexuality (44); Reckless spending (44)	Belligerence (75)	Distractibility (82)	More talkative (77)	Grandiose (27)	Hardly slept, not tired (38)					Impulsivity (22); Gross overactivity (22)	Restlessness (39)
	Flight of ideas (67)			Hyperactive (26)	Grandiose (27)						Gross overactivity (23)

All  % are reported to the nearest integer

^a^ K-SADS symptom ratings

^b^ Study reported only 3 key symptoms of mania; euphoria was not reported by any child participant

^c^ Study reviewed most common symptoms of BD, which also included ratings of some depressive features

**Table 3:** Rank order of severity of manic symptoms by age group (symptoms are reported as mean score or  %)

McElroy et al. (1997)^a^		Birmaher et al. (2009)^b,c^		Safer et al. (2012)^a^	
Mean ± SD		Mean ± SD		POMP^d^ (%)	
Adolescent	Adult	Child	Adolescent	Child/adolescent	Adult
Increased motor activity (2.5 ± 1.4)	Bizarre/grandiose thought content (3.2 ± 1.4)	High energy (4.3 ± 1.4)	High energy (4.8 ± 1.0)	Irritability (17)	Grandiosity (16)
Delusions (2.1 ± 1.7)	Delusions (2.7 ± 1.4)	Increased motor activity (4.3 ± 1.2)	Decreased need for sleep (4.5 ± 1.7)	Aggression (15)	Rapid speech (16)
Bizarre/grandiose thought content (2.1 ± 1.9)	Thought disturbance (2.2 ± 1.6)	Irritability (4.1 ± 1.5)	Elation (4.4 ± 1.0)	Rapid speech (15)	Irritability (14)
Thought disturbance (1.4 ± 1.6)	Sleep disturbance (1.9 ± 1.2)	Mood lability (4.1 ± 1.1)	Increased motor activity (4.3 ± 1.1)	Grandiosity (10)	Motor activity (10)
Sleep disturbance (1.3 ± 1.3)	Increased motor activity (1.8 ± 1.4)	Elation (3.9 ± 1.2)	Accelerated speech (4.2 ± 1.1)	Motor activation (9)	Elevated mood (9)
Increased goal directed/aggressive behaviour (0.8 ± 1.1)	Increased goal directed/aggressive behaviour (1.1 ± 1.2)	Accelerated speech (3.9 ± 1.2)	Poor judgement (4.0 ± 1.6); Racing thoughts (4.0 ± 1.3)	Elevated mood (9)	Aggression (9)

N.B. The range of possible scores for YMRS and K-MRS items differ, so mean scores are not directly comparable for similar symptoms

^a^ Young Mania Rating Scale (YMRS); ^b^ K-SADS Mania Rating Scale (K-MRS)

^c^ Most items are from the YMRS, but ‘delusions’ and ‘bizarre/grandiose thought content’ were from the Scale for Assessment of Positive Symptoms

^d^ POMP = Percent of maximum possible score; All  % are reported to the nearest integer

The symptoms as described were then put in rank order (with the most common or severe symptom ranked first) and tabulated. (It is important to note that the authors did not make any modification to the reported symptoms or items at this stage and, for example, as the construct grandiose/bizarre thought content is reported as a single symptom in the assessments reported in several studies, we retained that descriptor of presenting phenomenology in our review). If two or more items in an assessment scale occurred at the same frequency or had the same mean level of severity, we report both items (as they have an equal ranking). Any uncertainties on how to interpret the description or ranking of a symptom reported in the original data paper were resolved by consensus (JS and FR).

Having examined the reporting of the frequency and severity of symptoms as reported in eligible studies, it was noted that the studies showed heterogeneity in the assessment tools used, and most methodologies were rated as modest or lower quality. Also, there were only a small number of relevant publications available, especially for comparisons of severity of symptoms. As such, it was clear that it was not appropriate to use meta-analytic or other statistical approaches to the pooled data, and so we decided to use a simple strategy to give an insight into the distribution of manic symptoms in different age groups based on the rankings obtained for frequency and severity. First, we allocated each rank a numerical score (1st rank = 6; 2nd rank = 5, etc.). The total score for each manic symptom was calculated (as a composite of the ranking scores of frequency and severity for all studies), and the symptoms were then arranged in the descending order (using this score). We selected the ten highest scoring items and examined the description of each item to establish if there were duplications in regards to overlapping or similar symptoms in the list (e.g. some scales separate activity and energy, some combine them in a single item, etc.). Similar or overlapping symptoms were grouped into single items (e.g. irritability and aggression were collapsed into a single item; activity and energy were collapsed into a single item), and the ranking scores were adjusted accordingly. This strategy produced a final list of the five most common manic symptoms reported across studies. We then examined the ranking scores for each symptom by age group (expressing this as a weighted  %). It is emphasised that results offer a graphical representation of symptom distributions by age group (we did not apply statistical significance tests as this was deemed inappropriate). Our goal is simply to establish which symptoms are more prominent (in terms of frequency and/or severity) in each age group compared to the total sample included in the review.

## Results

As noted in Fig. 1, nine studies met eligibility criteria for inclusion in the review. The CASP assessment revealed that three studies achieved good-to-high-quality ratings (Findling et al. 2001; Birmaher et al. 2009; Chan et al. 2011), three were rated as good-to-modest (McElroy et al. 1997; Lazaro et al. 2007; Safer et al. 2012), whilst three studies achieved lower scores, suggesting some methodological weaknesses (Ballenger et al. 1982; Jerrell and Shugart 2004; Song et al. 2010).

As shown in Table 1, the studies were published over a 30-year period. Six of the nine studies were from the USA. Sample sizes ranged from 21 to 1106; in five studies, most of the participants were male. Three studies reported data from inpatients only and three from outpatients only. Five studies focused on BD-I cases, and the remaining studies included mixed samples of BD-I, BD-II and BD-NOS cases.

Fifty-six percent of the studies (5 out of 9) compared the symptoms of (hypo)mania in children versus adolescents (Findling et al. 2001; Lazaro et al. 2007; Birmaher et al. 2009; Song et al. 2010; Chan et al. 2011). Two studies combined children and adolescents into one group (minimum age 7 years; maximum age 17 years) and compared the younger group with adults (Jerrell and Shugart 2004; Safer et al. 2012). The other two studies compared groups of adolescents (maximum age ranged from 18 to 21 years) to adults (minimum age varied from 19 to 30 years) (Ballenger et al. 1982; McElroy et al. 1997).

Tables 2 and 3 report the findings regarding the rank order of symptoms based on the frequency or severity rating of each item. Four of seven studies identified irritability or irritability and aggression as the highest ranking symptom in the youngest age group assessed (either children alone or a group comprising children and adolescents). The two highest ranking symptoms in the seven studies that included an adolescent group were increased activity/energy, closely followed by elated/euphoric mood (Ballenger et al. 1982; McElroy et al. 1997; Findling et al. 2001; Lazaro et al. 2007; Birmaher et al. 2009; Song et al. 2010; Chan et al. 2011). One study (Ballenger et al. 1982) compared adolescents and adults and found that grandiosity was more common in the adolescent group and pressured speech was ranked highest in the adult group; decreased sleep was a frequent symptom for both age groups. McElroy et al. (1997) also compared adolescents with adults; the study reported that increased motor activity was the highest ranking symptom in the former compared to bizarre/grandiose thought content in the latter; psychotic symptoms (namely delusions) were the second most severe symptom reported in both groups.

**Fig. 2:**
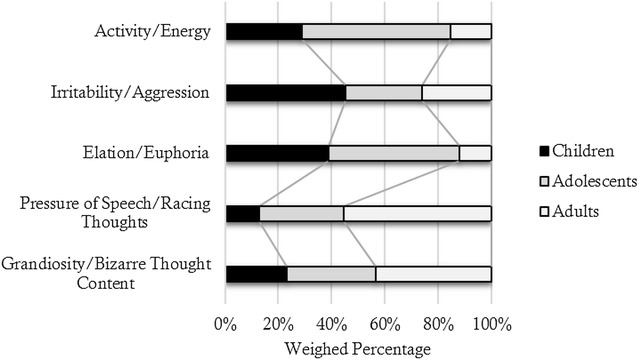
Schematic representation of symptom patterns across age groups (based on a weighting of derived from the frequency and severity of each symptom)

Figure 2 shows the data on symptom distributions (using a composite ranking of frequency and severity) reported as weighted percentages by age groups. As shown, there are some variations in symptom patterns by age, with irritability/aggression being the most prominent feature of childhood BD and activity/energy is the most prominent in adolescent BD; the second most prominent symptom is both these age groups is elated/euphoric mood. In adult BD, the two most prominent symptoms are those associated with changes in cognition (namely speed of thinking as described by pressure of speech and racing thoughts; and content of thinking as described by grandiose or bizarre ideas).

## Discussion

The aim of this systematic review was to explore whether there are any differences in the phenomenology of (hypo)manic episodes reported in studies that compare symptoms across groups with different ages of onset. As we examined both the frequency and severity of symptoms, this approach also offered some insights into whether the instruments used to measure the symptoms influence the patterns of symptoms observed. Before discussing our findings, it is important to note that there were limitations in achieving these aims of this review. Firstly, the studies defined the boundaries of three age groups in different ways. For instance, Ballenger et al. (1982) considered adolescence to extend to the age of 21, whereas most of the other studies used an upper age limit of 18. Secondly, the relative lack of eligible studies and the different compositions (some combining children and adolescents) and sizes of the age groups meant that we were only able to obtain data on about 2000 cases (268 children, a combined subsample of 540 children and adolescents, 265 adolescents and 933 adults). The limited number of cases per age group in the reviewed studies is further complicated by the heterogeneity in the range of BD subtypes included and timing of assessment of symptoms. Also, none of the eligible studies used the revised criteria for the diagnosis of BD as written in the DSM-5, which now incorporate activity and energy alongside mood change as the criterion A symptom for (hypo)mania (APA 2013). Thirdly, six studies were conducted in the USA, where approaches towards the diagnosis of BD in children has tended to differ from some, but not all, other parts of the world (Dubicka et al. 2008; Douglas and Scott 2014; James et al. 2014). Most importantly, despite the level of interest expressed in the phenomenology of BD and whether it is different in children, very few studies exist that directly compare the symptoms of paediatric BD with adolescent-onset or adult-onset BD; and even fewer studies use samples recruited in the same location and/or at the same time. These issues are relevant as diagnostic procedures and practices show both geographic and temporal trends (Mackin et al. 2006; Moreno et al. 2007).

The most significant finding of this review is that only nine publications met eligibility criteria for inclusion, and these studies used a range of methodologies and approaches to symptom assessment. Some relied on reviews of case notes and, even if this retrospective reporting of data was reliable, some of the studies were hampered by focusing on relatively few symptoms of mania [e.g. Lazaro et al. (2007) only examined three symptoms]. Others included ratings of additional symptoms of BD, such as depression (e.g. Song et al. 2010), without specifying if these occurred within a manic episode (suggesting the possibility of mixed states) or outside the manic phase. Also, studies of the frequency of manic symptoms often used different tools, some of which did not even include symptoms that are deemed core features of mania. Of those studies relying on severity scores, the use of different rating scales (e.g. the YMRS and K-MRS), made cross-study comparisons difficult, as the scales do not include identical sets of symptoms, or they give different weightings to the same manic symptoms. For instance, irritability in the YMRS (Young et al. 1978) is rated 0–8, whilst most other symptoms are rated 0–4. However, on the K-MRS (Kaufman et al. 1997), all but one item are rated 1–6 (distractibility is rated 1–5). Furthermore, some scales use composite ratings for activity and energy or for irritability and aggression, grandiose/bizarre thinking, etc. As such, it is likely that the different approaches to assessment by the original researchers have influenced the findings of this review. We tried to overcome some of these problems using rank-ordering the phenomena, but it is emphasized that most findings from the synthesis of pooled data must be treated with caution.

It was notable that none of the studies in this review directly compared a ‘child only’ group to an ‘adult only’ group. This is surprising, given the debate about the similarities or differences in symptoms profiles and nature of BD in these two age groups. Safer et al. (2012) and Jerrell and Shugart (2004) compared a group comprised children and adolescents with an adult group. Their findings suggest irritability is more prominent in the younger age group compared to the adults. Both studies included in- and out-patients, which may indicate that cases were at a more severe end of the spectrum than in some other studies; however, Safer et al. (2012) did not recruit all the participants at the same time, and the cases in each group were not assessed by the same clinicians, which introduces potential sources of bias.

The pattern of manic symptoms in children compared to adolescents varied across studies and by assessment procedure. Figure 2 identifies that irritability/aggression was more prominent in children diagnosed with BD compared to adolescents, which offers some support to previous findings which suggest that irritability is frequent and severe in paediatric and juvenile BD (e.g. Tillman and Geller 2007; Soutullo et al. 2009). A recent meta-analysis of 20 studies (Van Meter et al. 2016) suggested that irritability was the second most prevalent symptom of mania (77%) in childhood (interestingly, that meta-analysis found that increased energy was the most common symptom). However, Van Meter et al. (2016) took a different approach to study selection than used in the current review, and assessed symptom distribution within paediatric BD using a wider range of studies, most of which did not compare the distribution of symptoms across childhood-, adolescent and adult-onset groups. As such, the reviews offer complementary rather than competing views of the phenomenology of (hypo)mania in childhood-onset cases of BD.

Whilst the finding regarding irritability in younger age groups is of interest, it is important to note that irritability cannot be regarded as a specific indicator of bipolarity. For instance, periods of irritability can be part of normal development in young children and adolescents (Pataki and Carlson 2013), so irritability on its own may not indicate any underlying disease process. In contrast, persistent irritability or distractibility might be a feature of other mental disorders, such as ADHD, or of underlying organic brain disease, rather than part of a manic syndrome (Vidal-Ribas et al. 2016). This is especially relevant to this review, as many studies of prepubertal BD in the literature suggest comorbidity rates with ADHD that exceed 50% (range 30–95%) (e.g. Faraone et al. 1997; Biederman et al. 1996; Bernardi et al. 2010). Indeed, some reviews question whether it is possible to reliably differentiate ADHD from BD in children, or whether ADHD may be misdiagnosed as BD [e.g. Skirrow et al. (2012)]. We were not able to determine whether the symptom of irritability/aggression recorded in the studies in this review included only episodic phenomena or encompassed more chronic presentations. However, clarification is needed, as Leibenluft et al. (2003) highlights that there is a degree of uncertainty about how to classify some of these cases and Meyer et al. (2011) suggested that child psychiatrists should perhaps rely more on what are considered prototypical manic symptoms such as increased energy or decreased need for sleep when diagnosing BD in children. Interestingly, the DSM-5 now includes a separate category of Disruptive Mood Dysregulation Disorder, which is likely to lead to revisions in how some cases with presentations dominated by irritability being re-diagnosed.

Finally, whilst the primary focus of this review was to examine if comparative studies can shed light on the phenomenology of childhood-onset BD versus other age groups, the findings regarding the most prominent symptoms of adolescent and adult BD are also worthy of comment. The identification of activity/energy as a primary symptom in these clinical studies of adolescents confirms previous reports by Merikangas et al. (2011) derived from large-scale community-based cohort studies. To the best of our knowledge, the finding that cognitive symptoms (speed and content of thought) are more prominent in adults compared to younger age groups has not been reported previously. Whilst it is possible that this is an artefact of the weightings procedure used in this review to allow cross-study comparison of symptoms, the finding is worth highlighting. Part of the explanation for cognitive symptoms of mania being more marked in adults compared to children is that children may be less able to express their experiences or ideas verbally, the assessment procedure may not have been sufficiently sensitive to detect some of the cognitive changes in children, or the symptoms may have been attributed to a comorbid condition, etc. However, this explanation might not extend to the difference between adults and adolescents. As such, this (and our other findings on symptom patterns across age groups) warrant further research that applies more sophisticated approaches to the assessment of symptoms and their differential contribution to the presentation of (hypo)mania, such as item response theory (e.g. Weinstock et al. 2009).

## Conclusions

To the best of our knowledge, this is the first attempt to synthesize data from studies that directly compare symptoms of BD across groups defined by age. Irritability is a striking feature of mania in groups that include children (children only or children and adolescents) who were diagnosed with BD. However, given the debates regarding the similarities or differences between adult-pattern and childhood onset BD, it is disappointing that no study makes a direct comparison of the phenomenology observed in children and adults. Other findings of note in this review are the sparsity of eligible high-quality studies, the lack of geographical spread in available studies (leading to a bias towards studies undertaken in the USA), the failure of studies of phenomenology to fully account for the impact of comorbidity on symptom ratings and the methodological heterogeneity. Therefore, we conclude that systematic research on this topic is still required to answer important clinical questions about the presentation or evolution of (hypo)mania across different age groups, which is an issue that has implications not only for day-to-day practice, but also for research on the classification of mental disorders.

## Appendix: List of search terms used to identify publications

**Table:**

Group 1	Group 2	Group 3
Mani*	Young adult	Phenomeno*
Hypomani*	Kid*	Symptom*
Bipolar disord*	Adolescen*	Psychopathol*
Mani depress*	Teenage*	
Bipolar*	Youth	
	Child*	
	Post-pubertal	
	Underage*	
	Juven*	
	Universit*	
	School*	
	Student*	
	College*	
	Young pe*	
	Prepubertal	
	Paediatric	
	Pediatric	

## Abbreviations

ADHD: attention deficit hyperactivity disorder
APA: American Psychiatric Association
BD: bipolar disorders
BD-NOS: bipolar disorder, not otherwise specified
CASP: Critical Appraisal Skills Programme
DSM: diagnostic and statistical manual of mental disorders
ICD: International Classification of Diseases
K-SADS: Kiddie-Schedule for Affective Disorders and Schizophrenia
K-MRS: K-SADS Mania Rating Scale
NK: not known
POMP: Percent of Maximum Possible Item Score
PRISMA: preferred reporting items for systematic reviews and meta-analyses
SD: standard deviation
YMRS: Young Mania Rating Scale
